# Survey of Integrative Treatment Practices of Korean Medicine Doctors for Cervical Disc Herniation: Preliminary Data for Clinical Practice Guidelines

**DOI:** 10.1155/2019/2345640

**Published:** 2019-07-31

**Authors:** Hee Seung Choi, Yoon Jae Lee, Me-riong Kim, Jae-Heung Cho, Koh-Woon Kim, Eun-Jung Kim, In-Hyuk Ha

**Affiliations:** ^1^Jaseng Hospital of Korean Medicine, Seoul, Republic of Korea; ^2^Jaseng Spine and Joint Research Institute, Jaseng Medical Foundation, Seoul, Republic of Korea; ^3^Department of Korean Rehabilitation Medicine, College of Korean Medicine, Kyung Hee University, Seoul, Republic of Korea; ^4^Department of Acupuncture & Moxibustion, College of Korean Medicine, Dongguk University, Gyeongju, Republic of Korea

## Abstract

A survey study was conducted in Korean medicine doctors who provide nonsurgical integrative treatment for cervical disc herniation (CDH) at spine-specialty hospitals to assess usual treatment practices, diagnosis and treatment methods, and related adverse events for CDH. The questionnaire was jointly developed by clinical experts and methodology experts and was administered to 197 Korean medicine doctors (response rate: 84.9% (n = 197/232)) practicing at spine-specialty Korean medicine hospitals for analysis of general sociodemographic information, practice patterns of CDH including diagnosis and treatment strategies, CDH prognosis, and treatment safety. The average clinical experience of respondents was 9.3±6.4 years, and 4.0±1.8 weeks were regarded to be needed for CDH pain to decrease by 50% and 9.1±3.4 weeks to decrease by 80%. Eight-Principle Pattern and Meridian System Identification were the most commonly used Korean medicine syndrome differentiation methods, and CDH was most often considered to be a result of Qi stagnation and Blood coagulation. The Spurling test was reported to be important in physical examination, and magnetic resonance (MR) images were mostly used for diagnosis and treatment of CDH of various diagnostic tools. Treatment mainly consisted of a nonsurgical, integrative multimodal approach comprising acupuncture, pharmacopuncture, herbal medicine, and Chuna manual therapy. Shinbaro pharmacopuncture and Chungpa-jun, which are well-established herbal treatments supported by evidence, were considered to be of high importance in CDH treatment. With regard to safety, acupuncture was considered to be the safest, while bee venom pharmacopuncture was of highest concern due to potential hypersensitivity. This study is the first report to investigate current practice patterns and approach of Korean medicine doctors to CDH treatment. This data may be of significance to Korean medicine doctors in drawing clinical guidelines and conducting randomized controlled trials (RCTs) to generate high-level evidence on the effectiveness of nonsurgical integrative medicine treatments for CDH.

## 1. Introduction

Cervical disc herniation (CDH) is a condition where inflammation related to the herniated disc irritates the cervical nerves and/or compresses the nerve root, incurring pain or numbness of the upper extremity that may radiate extensively to the neck, shoulder, hand, and fingers [1, 2]. It was reported that 1.79 out of 1,000 United States military members developed CDH every year between 2000 and 2009 [3], and a recent study reported that 107.3 men and 63.5 women out of 100,000 individuals develop cervical radiculopathy each year [4]. According to the Healthcare Big Data Hub provided by the Korean Health Insurance Review & Assessment Service (HIRA), 1,939,400 patients presented with CDH in 2016, and national health insurance expenditure totaled 316 billion Korean won. CDH ranked high at 15^th^ place in reasons for admission to Korean medicine hospitals and 9^th^ in total national health insurance expenses by illness. These statistics collectively showcase the extent to which CDH impacts the population and the high frequency of use of Korean medicine for CDH treatment [5].

While early surgery for CDH has been shown to hold the advantage of swift pain alleviation, evaluation of overall patient outcomes has revealed that the long-term difference between surgical and nonsurgical treatments is nonsignificant [6], and the optimal timing for surgery is proposed to be after at least 6 to 8 weeks of nonsurgical treatment and only in cases of persistent pain [7, 8]. Nonsurgical treatment is recommended as first-line treatment for pain management in the absence of emergency surgery indications, and while conventional treatments such as medication and injections are extensively used for pain relief and improvement of quality of life [9, 10], patient interest in integrative medicine approaches for CDH treatment is increasing [11], partly due to the limited evidence for effectiveness of conventional treatment and associated adverse events [7]. These data support the practice of low prioritization of surgery in decision-making for CDH and highlight the significance of timely utilization of nonsurgical treatments within an integrative medicine model for effective pain management and favorable patient prognosis.

Korea operates a specialty hospital system where the Minister of Health and Welfare designates hospitals that perform high-proficiency medical procedures for specific diseases or medical specialties as selecting specialty hospitals (Article 3, Section 5 of Korean medical law). Spine-specializing Korean medicine hospitals offer various nonsurgical Korean medicine treatments such as acupuncture, herbal medicine, Chuna manual therapy, and pharmacopuncture, and while the effects of treatment have been attested to in various studies [12–14], few studies on nonconventional treatments including Korean medicine have been conducted with specific focus on CDH treatment as diagnostic imaging with magnetic resonance imaging (MRI) or computed tomography (CT) scans are required to be diagnosed with CDH, in which tools may not be readily available at most Korean medicine facilities [15]. There is also a continued lack of data on treatment trends and healthcare provider and stakeholder dispositions toward nonsurgical integrative treatment. The aim of this study was to investigate treatment trends, diagnostic methods, and significance of various nonsurgical integrative medicine treatment modalities through survey of Korean medicine doctors (KMDs) specializing in CDH treatment. As options, dosage, and prognosis for CDH treatment would differ from those for nonspecific neck pain, current practice patterns and clinical experience of KMDs who practice at spine-specialty Korean medicine hospitals and treat CDH on a regular basis should help provide a basis for evidence-based clinical guidelines on neck pain and especially CDH to physicians, researchers, and health policy makers with the aim of facilitating implementation of clinical guidelines into actual practice.

## 2. Materials and Methods

### 2.1. Questionnaire Development

**Figure 1:**
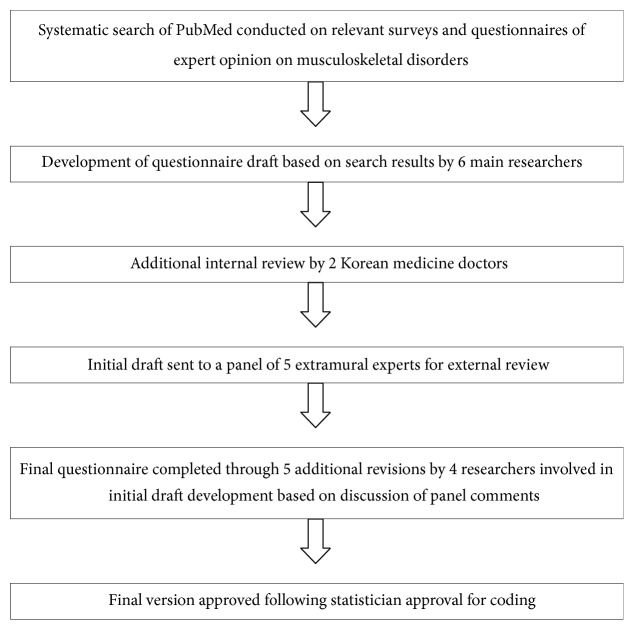
Questionnaire development process.

The final distributed questionnaire was developed through modification of the initial questionnaire following consultation and discussion with clinical and methodology experts, and the questionnaire accordingly underwent subsequent revisions. Details of the overall questionnaire development process can be found in previous survey studies conducted on lumbar disc herniation (LDH) and lumbar spinal stenosis [16, 17]. In summary, 6 KMDs who received 6 years of professional medical education (consisting of 4 Korean medicine rehabilitation specialists with average 10+ years of clinical experience and 2 Korean medicine rehabilitation residents in training with 3+ years of clinical experience) employed at a spine-specialty Korean medicine hospital that was the study setting participated in development of the questionnaire draft. A systematic search of PubMed was conducted using such terms as “herniated disc, survey, questionnaire, clinical decision, and consensus” to heighten objectivity and external validity, and an initial draft was established based on the search results; this draft was further revised following review by 2 other KMDs employed at the same hospital. The initial draft was then sent electronically to a panel of 5 extramural experts for their written opinion. The panel comprised a Korean medicine rehabilitation professor at a Korean medicine university, a Korean medicine rehabilitation professor at a specialized Korean medicine graduate school and the president of an academic society of spine manipulation, a researcher at the Korean Institute of Oriental Medicine (KIOM, a government-funded research center for Korean medicine and subsidiary organization of the Korea Research Council of Fundamental Science and Technology under the Korean Ministry of Science Information & Communication Technology and Future Planning), a researcher at KIOM and acupuncture specialist, and a methodologist and former researcher of the National Evidence-based healthcare Collaborating Agency (NECA). Panel comments and suggestions to be considered for revision were collected, and 4 researchers involved in initial draft development convened for discussion. The final questionnaire was completed through 5 additional revisions. The final version was printed following statistician approval for coding (Figure 1).

Doctors of Korean medicine employed at Korean medicine hospitals, which included all spine-specialty Korean medicine hospitals as designated by the Ministry of Health and Welfare at the time of survey distribution (Jaseng Hospital of Korean Medicine Gangnam branch, Jaseng Hospital of Korean Medicine Bucheon branch, Jaseng Hospital of Korean Medicine Daejeon branch, and Mokhuri Neck & Back Hospital), were investigated.

The survey was administered on August 28^th^, 2016, at an internal conference for KMDs at spine-specialty Korean medicine hospitals. The questionnaire was delivered by post or e-mail to doctors who were not present at the conference. The survey was completed without receiving signatures to ensure anonymity, and sufficient explanations of the purpose, development procedure, and questionnaire answering methods were provided to minimize potential misinterpretation and errors in the answering process. Informed consent regarding protection of personal information and the use of results for academic means were obtained. The study was approved by the Institutional Review Board (IRB) of Jaseng Hospital of Korean Medicine (JASENG 2016-08-005). The English language version of the complete questionnaire is provided as a Supplementary File [Supplementary Materials [Supplementary-material supplementary-material-1]].

### 2.2. Data Entry

A designated statistician designed the data entry form using Microsoft Office Excel version 14.0 (Microsoft®, Redmond, WA, USA) and trained two independent researchers who were not involved in questionnaire development or study publication on the method of data entry. Following initial data entry, the statistician inspected the dataset and marked missing and ambiguous entries to be delivered to a Korean medicine physician research investigator. Duplicate answers for questions that did not allow for multiple answers were processed as missing data. In cases where the intent of the answer was unclear due to use of Korean medicine or obscure terminology, the term in question was marked in a different color to denote its ambiguity and reported to a Korean medicine physician investigator in an effort to reduce errors in data analysis.

### 2.3. Method of Analysis

Regarding descriptive statistics, continuous variables are presented as mean ± standard deviation (SD), and categorical variables as frequency and percentage (%). Most categorical variable items allowed for multiple answers, and as multiple responses were given as such, separate analyses were performed for items of highest ranking. Likert scales were converted and assessed as continuous variables in analysis. All statistical analyses were conducted using SAS version 9.4 (SAS institute Inc., Cary, NC, USA).

## 3. Results

### 3.1. Sociodemographic Information

The response rate was 84.9% (n = 197/232). The survey respondents consisted of a total 197 doctors of Korean medicine and were composed of 183 male and 14 female doctors with an average age of 35.4±7.3 years. The average length of clinical experience was 9.3±6.4 years, and 118 of the doctors (60.4%) had extensive clinical experience of ≥10 years. Of respondents, 91.4% worked at secondary medical facilities (secondary facilities including Korean medicine hospitals hold 30≤ and <500 beds for inpatient care), and 44.4% reported bachelor's degrees, 27.0% master's degrees, and 28.6% Ph.D. degrees as the highest level of education attained. A total 56.5% of respondents had completed medical specialty training, of which 41.6% were specialists of Korean Medicine Rehabilitation, 23.0% were specialists of Korean Acupuncture and Moxibustion Medicine, and 21.1% were specialists of Internal Korean Medicine. Of the surveyees who had received extracurricular education sessions outside formal university education for Korean medicine, 79.7% had attended courses provided by the Korean Society of Chuna Manual Medicine for Spine & Nerves (Table 1).

### 3.2. Practice Patterns

The respondents reported that they treated an average 15.9±12.8 CDH patients/day and that they saw each patient for 2.1±1.1 sessions/week for an average duration of 24.0±16.2 minutes/session. The main treatment methods were acupuncture (87.3%), pharmacopuncture (87.8%), herbal medicine (82.2%), and Chuna manual therapy (81.8%), all of which were reported at high percentages of over 80%, and cupping (76.1%), physical therapy (56.3%), bee venom pharmacopuncture (53.3%), and Doin conduction exercise (43.1%) use were also reported at high frequencies. Surveyees replied that it took an average of 4.0±1.8 weeks of outpatient sessions for 50% decrease in pain and about double that time at 9.1±3.4 weeks, to reach a decrease in pain severity of 80% (Table 2).

### 3.3. Diagnostic Tests, Examinations, and Prognosis

The factors considered to influence patient prognosis most were reported to be clinical symptoms (69.0%), radiologic imaging test results (55.8%), onset duration and cause (39.1%), past medical history (32.0%), and patient awareness of and attitude towards disease (28.9%), in decreasing order (Table 3). The most commonly referenced examinations were all imaging tests including magnetic resonance (MR) images (99.5%), X-rays (97.5%), and computed tomographies (CTs) (69.0%), and they were preferred to blood tests, electromyograms (EMG) and digital infrared thermal imaging (DITI). Remarkably, MR images were chosen by 82.7% of respondents as the most significant test. The most common focus for magnetic resonance imaging (MRI) results was the degree of nerve compression by the herniated disc (87.3%), degree of disc herniation (71.6%), and correlation of clinical symptoms with disc herniation as shown on MRI (60.4%). Physical tests of high significance were reported to be the Spurling test (85.8%), followed by the foraminal compression test (44.2%), muscle strength grading (36.5%), Adson's test (28.4%), and the distraction test (27.4%). When asked to choose the single most important physical test, 69% of respondents selected the Spurling test over other physical examinations (Table 4).

### 3.4. Korean Medicine Syndrome Differentiation

The participants responded that the Eight-Principle Pattern Identification (69.5%), Meridian System Diagnosis (65.0%), and Etiological Factor Syndrome Differentiation (48.2%) were the most frequently applied syndrome differentiation methods for diagnosis and treatment. Korean medicine diagnoses of high relevance for CDH patients were considered to be stagnation of Qi and coagulation of Blood (88.3%), lack and deficiency of Liver and Kidney with Exopathogens (53.3%), and deficiency of Qi and Blood (51.3%) (Table 4).

### 3.5. Treatment Methods

The most effective treatment methods for CDH were regarded to be herbal medicine, Chuna manual therapy, bee venom pharmacopuncture, pharmacopuncture, and acupuncture. Pharmacopuncture, bee venom pharmacopuncture, herbal medicine, Chuna manual therapy, and acupuncture were construed to be most effective for short-term treatment (8 weeks), in descending order, and, for long-term treatment (1 year), herbal medicine, pharmacopuncture, Chuna manual therapy, bee venom pharmacopuncture, and acupuncture were reported as most effective (Table 3).

The most effective herbal medicines were considered to be Chungpa-jun (31.3%), Seokyung-tang (28.4%), Gamihwalhyul-tang, and Galgeun-tang (8.7%) in multiple answers, and Chungpa-jun (81.1%) was selected as the primary herbal medicine of choice, which may be taken to be indicative of its clinical importance. The most commonly used Chuna techniques were supine cervical JS distraction correction technique (82.7%), supine cervical distraction technique using both hands (44.7%), supine cervical correction technique (35.5%), and prone cervical distraction method (29.4%) (Table 5).

Preferred acupuncture point selection methods were the anatomical region causing symptoms (71.1%), pathological area as identified by diagnostic imaging (59.9%), tender points or trigger points that reproduce pain upon pressure (53.3%), Ashi points (43.7%), and effective points as determined from clinical experience (33.5%). An average of 10.0±3.5 needles were used on each patient per session to a depth of 2.1±0.9 cm for 13.3±2.5 minutes. Electroacupuncture was administered in most cases (93.5±15.8%) (Table 6), and the most commonly targeted acupuncture points were GB20, GB21, GV16, LI11, SI03, and LI04, in descending order of frequency. While effective acupuncture methods were identified as Ashi point acupuncture (95.9%), motion style acupuncture treatment (MSAT) (87.8%), and symptomatic point selection (53.3%) (when multiple responses were allowed), the single acupuncture methods identified as most significant were of a slightly different order: MSAT (45.7%), Ashi point acupuncture (34.5%), and symptomatic point selection (14.7%), suggesting that MSAT is the most important acupuncture method for CDH in this population of Korean medicine physicians (Table 5). Common acupuncture points used for pharmacopuncture injection were GB20, GB21, and GV16, applied with an average needle length of 1.3 cm for anterior needling and 2.7 cm for posterior needling, injecting 1.1 cc for the anterior and 2.6 cc for posterior regions on average per session (Table 6). Preferential pharmacopuncture solutions included Shinbaro, bee venom pharmacopuncture, and Hwangryunhaedok-tang when multiple answers were allowed, but the single most preferred pharmacopuncture treatment was Shinbaro (70.6%), followed by bee venom (9.6%) and Hwangryunhaedok-tang (4.1%), which suggests a stronger preference for Shinbaro pharmacopuncture in limited use.

### 3.6. Safety

Survey data on KMD perception on the safety of frequently used treatment methods were collected using a 7-point scale as follows: 1=very unsafe, 2=unsafe, 3=somewhat unsafe, 4=neither safe nor unsafe, 5=somewhat safe, 6=safe, and 7=very safe and showed that KMDs considered bee venom to hold the lowest level of safety out of surveyed treatments at 4.7±1.3 and acupuncture to be the safest form of treatment at 6.3±0.8 out of the provided items of herbal medicine, Chuna manual therapy, bee venom pharmacopuncture, acupuncture, and other treatments (Table 7). Adverse events taken into account during treatment were reported to be mainly allergic symptoms such as itching and rashes, anaphylaxis (bee venom pharmacopuncture), pneumothorax (acupuncture), aggravation of pain (acupuncture, Chuna manual therapy, and pharmacopuncture), hemorrhages and blood vessel injury (acupuncture, pharmacopuncture), disorders of the digestive system, and change in bladder and bowel habits (herbal medicine).

## 4. Discussion

Although surgery should be duly considered in CDH cases demonstrating progressive neurological symptoms, motor weakness, and sensory deficit, or cases of persistent pain nonrespondent to conservative treatment [18, 19], conservative treatment for at least six to eight weeks is indicated prior to such surgical considerations [7, 10]. Conservative treatment itself has been purported to be quicker than early surgical interventions in reducing pain and initiating self-perceived recovery [20]. However, conservative treatment generally lacks quantitative evidence [7, 21], and findings supporting its pain control and functional recovery effects are somewhat limited [22]. Meanwhile, the effectiveness of integrative medicine for cervical radiculopathy and neck pain has been receiving wider recognition, giving lead to the recent surge of related studies [23–26]. Unfortunately, most systematic reviews on integrative medicine for CDH and cervical radiculopathy report studies of low or moderate methodological quality [25], demonstrating the need for more definitive evidence generated through well-designed, rigorous clinical trials.

Korea employs a dual medical system that utilizes both conventional and Korean medicine. Spine-specialty Korean medicine hospitals as certified by the Korean Ministry of Health and Welfare treat various musculoskeletal disorders using nonsurgical integrative medicine techniques including acupuncture, herbal medicine, Chuna manual therapy, and pharmacopuncture [13–15, 27] and operate an integrative treatment model collaboratively with conventional medicine for effective diagnosis (e.g., X-rays, MR images, CT, DITI, and clinical laboratory tests) and pain control means (e.g., oral analgesics, epidural injections, and physiotherapy). This study aimed to establish and propose a model of integrative usual care that may be of reference to CDH practitioners and researchers through concurrent and comprehensive reporting of the clinical experience and practice patterns for CDH of KMDs practicing at spine-specialty Korean medicine hospitals.

In the spine-specialty Korean medicine hospitals where the respondents practice, MRI examinations are frequently conducted in spinal disorder patients of moderate or severe conditions, and the treatment is initiated after confirming “cervical disc herniation.” Therefore, the “cervical disc herniation” patients that the current survey respondents (KMDs) treat can be conjectured to differ from neck pain patients with radicular pain diagnosed with “cervical spondylosis radicular type” who may or may not have MRI readings. The respondents of this study treated an average of 15.9±12.8 CDH patients/day and used nonsurgical integrative medicine treatment methods encompassing acupuncture, pharmacopuncture, herbal medicine, and Chuna manual therapy. The most influential factors for determining patient prognosis were identified as clinical symptoms and radiologic tests, especially MRIs. Of physical tests, although the Spurling test has been reported not to be particularly sensitive, it has relatively high specificity [28] and was used frequently. The Korean medicine syndrome differentiations used in treatment were Eight-Principle Pattern Identification and Meridian System Diagnosis, and the most relevant Korean medicine classification of CDH was considered to be stagnation of Qi and coagulation of Blood. Herbal medicine, bee venom pharmacopuncture, Chuna manual therapy, pharmacopuncture, and acupuncture were recognized to be important treatment methods, and the importance of herbal medicine increased for long-term effects. Acupuncture point selection through pathologies confirmed by radiologic imaging and anatomical areas relating to pain was preferred to point selection according to traditional Korean medicine theory, while Shinbaro pharmacopuncture and Chungpa-jun were considered to be of high importance of Korean medicine treatment methods. These results point to the advantages of integrative medicine use coupling conventional medicine diagnostic practices with less invasive and more conservative Korean medicine treatment methods for safe and effective CDH treatment.

Compared to the 2015 LDH survey study [16], the number of participating KMDs increased, but the perspective on diagnosis and treatment of disc herniation was mostly similar. Meanwhile, though previous studies have suggested that medication treatment for CDH results in improvement at lower dosages than LDH [29], the current survey results implied that the duration of outpatient sessions necessary for pain reduction in CDH patients was similar at 4.0±1.8 weeks for 50% decrease to the 4.3±1.9 weeks required in LDH patients. Likewise, the length of outpatient treatment needed for pain reduction by 80% in CDH patients was 9.1±3.4 weeks and was comparable to the 9.6±3.5 weeks in LDH patients. Patterns of practice for CDH and LDH diagnosis and treatment including prognostic factors, the order of importance for medical examinations, Korean medicine syndrome differentiation, acupuncture point selection methods, and preferred pharmacopuncture and herbal medicine types were also similar. This study further verified that Chungpa-jun is used extensively for CDH which may be in reference to its anti-inflammatory, nerve regenerative, and bone and cartilage protective properties and clinical experience [30–32], rather than using more traditional treatment methods such as Hoesu-san and Gamiseokyung-tang based on Meridian System Diagnosis. In summary, although the KMDs who participated in this survey observed traditional Korean medicine theories such as Eight-Principle Pattern Identification and Meridian System Diagnosis in diagnosis of CDH patients, they also incorporated more modern approaches such as physical examination and MR images as recommended in conventional medicine guidelines [7] within the context of an integrative treatment model. This model of evidence-based integrative medicine was also shown to place emphasis on Chungpa-jun and Shinbaro pharmacopuncture use which contain GCSB-5 extracts, a compound of the key ingredients in Chungpa-jun [32].

Study Limitations. The largest limitation of this study is the generalizability of the results as the respondents consisted of a dominantly male Korean medicine specialist population aged ≤41 years; medical specialists comprise approximately 10% of the total KMD population [33]. Although the current responses may reflect expert opinion, the study is flawed in that the study population is biased since it is not fully inclusive of KMDs and their opinion and may thus introduce bias regarding the treatments generally used by KMDs due to noninclusion of the general population of Korean medicine doctors who treat CDH patients at local clinics on a community level. Still, the treatment modalities surveyed in this study are not exclusive to medical specialists, Korean medicine hospitals, or spine-specialty hospitals, and these results may be considered to hold significance in that it may provide valuable information on clinical experience in treatment of CDH to KMDs, researchers, and health policy administrators as it covers a wide range of nationwide Korean medicine doctors employed at spine-specialty Korean medicine hospitals designated as such by the Korean Ministry of Health and Welfare and as such comprehensively addresses the opinion of Korean medicine doctors who treat CDH patients at a high frequency with the use of diagnostic imaging such as MRIs. In addition, it is a survey centered on physician as opposed to patient-reported outcomes, and the results are susceptible to possible overestimation of treatment effects and risk of recall bias. However, despite these limitations, this analysis of clinical practice holds significance as the first study to report current practice patterns for CDH diagnosis and treatment by KMDs, which is essential for forming the evidence-based foundation upon which future guidelines can be established. Moreover, it included 1.6 times more respondents than the preceding LDH study. Previous survey studies of expert opinion also suffer the limitation of limited generalizability in practice scope and region (e.g., European neurosurgical trainees, spine and pain clinics in North Carolina, and physiotherapists specializing in manual therapy) [34–36]. In light of the fact that there is insufficient evidence on nonsurgical treatments for CDH with the exception of epidural steroid injections [7] and in line with reports that the benefits of cervical surgeries fall short of those of lumbar surgeries [37], this study is expected to contribute to the design of high-quality clinical trials as a concurrent report on nonsurgical treatments provided by KMDs.

## 5. Conclusion

Although the results of this study cannot be held to represent the general opinion of KMDs, it examined the practice patterns, diagnostic methods, and significance of various nonsurgical integrative medicine treatment modalities through survey of clinical KMDs who specialize in CDH treatment and are employed at spine-specialty Korean medicine hospitals in Korea. The results reflect the practice of prioritizing evidence-based CDH treatment combining high-precision integrative medicine diagnosis with traditional Korean medicine syndrome differentiation. The findings of this study illustrate the clinical practice patterns of KMDs and are expected to further contribute to strengthening the evidence base of nonsurgical integrative medicine for CDH.

## Figures and Tables

**Table 1:** Demographic characteristics of Korean Medicine doctor surveyees.

Factors	Mean±SD/n (%)
Age (years)	35.4±7.3

<34	86 (44.3)
<37	42 (21.6)
<41	29 (14.9)
≥41	37 (19.1)

Sex	

Male	183 (92.9)
Female	14 (7.1)

Clinical experience (years)	9.3±6.4

<8	77 (39.5)
<11	50 (25.6)
<15	35 (17.9)
≥15	33 (16.9)

Facility level of current affiliation^a^	

Primary clinic	17 (8.6)
Secondary medical facility	180 (91.4)

Highest academic degree	

Bachelor's	87 (44.4)
Master's	53 (27.0)
Ph. D.	56 (28.6)

Medical specialist training	

Yes (specialist)	109 (56.5)
No (general practitioner)	31 (16.1)
In training (resident)	53 (27.5)

Medical specialty (if applicable)	

Society of Korean Medicine Rehabilitation	67 (41.6)
Korean Acupuncture and Moxibustion Medicine Society	37 (23.0)
Society of Internal Korean Medicine	34 (21.1)

Additional extracurricular training^b^	

Korean Society of Chuna Manual Medicine for Spine & Nerves	157 (79.7)
Society of Korean Medicine Rehabilitation	55 (27.9)
Korea Pharmacopuncture Institute	37 (18.8)
Korean Acupuncture and Moxibustion Medicine Society	33 (16.8)
Korean Academy of Sports Oriental Medicine	31 (15.7)

^a^Primary clinics operate <30 beds for inpatient care.

Secondary medical facilities operate 30≤ and <500 beds for inpatient care and at least 4 outpatient departments including medical specialties.

^b^The curriculum in “extracurricular training” refers to the 6 years of Korean Medicine education provided at Korean Medicine universities or 4 years of postgraduate courses provided at a specialized Korean Medicine graduate school, a prerequisite for all certified KMDs.

**Table 2:** Clinical practice patterns of Korean Medicine doctors for cervical disc herniation treatment.

	Mean±SD/n (%)
Usage rate of treatment (multiple responses allowed)	

Pharmacopuncture	173 (87.8)
Acupuncture	172 (87.3)
Herbal medicine	162 (82.2)
Chuna manual therapy	160 (81.2)
Cupping	150 (76.1)
Physiotherapy (including electrotherapy, light therapy, and hydrotherapy)	111 (56.3)
Bee venom pharmacopuncture	105 (53.3)

Number of CDH outpatient consultations/day	15.9±12.8
Number of treatment visits/week per CDH outpatient	2.1±1.1
Average length of treatment required for 50% pain relief (weeks)	4.0±1.8
Average length of treatment required for 80% pain relief (weeks)	9.1±3.4

CDH, cervical disc herniation.

**Table 3:** Influence of factors in prognosis determination and importance of individual treatment method effects for cervical disc herniation.

Prognostic factors	Importance	Treatment methods	Short-term (8 weeks) treatment effects	Long-term (1 year) treatment effects
	Mean±SD		Mean±SD	Mean±SD
Clinical symptoms^a^	6.2±0.9	Herbal medicine	6.1±0.9	6.3±0.9
Radiological findings	5.9±1.0	Pharmacopuncture	6.3±0.8	5.9±1.0
Medical history	5.8±0.9	Chuna manual therapy	6.0±0.8	5.7±1.1
Patient perception of and attitude toward disorder	5.7±1.1	Acupuncture	5.9±1.0	5.7±1.1
Time from onset and cause of onset	5.6±1.1	Bee venom pharmacopuncture	6.1±0.9	5.5±1.2
Age	5.4±1.0	Moxibustion	4.0±1.3	4.3±1.4
Physical examination	5.2±1.2	Cupping	4.7±1.3	4.4±1.3
Personality and other psychological factors	5.2±1.2	Physiotherapy	4.9±1.2	4.5±1.3
Comorbidities	4.5±1.2	Doin conduction exercise	4.6±1.3	4.8±1.4
Korean medicine syndrome differentiation	3.9±1.4	Qigong, Tai Chi	3.3±1.5	3.7±1.7
Other (exercise habits, muscle mass, occupation, and posture)	6.0±0.0	Other	3.4±1.6	3.5±1.7

^a^Factor most frequently ranked 1^st^

(Importance: 1=not important at all, 2=unimportant, 3=somewhat unimportant, 4=neither important nor unimportant, 5=somewhat important, 6=important, and 7=very important.)

**Table 4:** Diagnostic tools most frequently used for cervical disc herniation and Korean Medicine syndrome differentiation of symptoms.

Factors	n (%)
Tests	

Magnetic resonance imaging (MRI)^a^	196 (99.5)
Simple X-ray	192 (97.5)
Computed tomography (CT)	136 (69.0)
Digital infrared thermal imaging (DITI)	19 (9.6)
Electromyogram	14 (7.1)

Main points of consideration in reading MRI images	

Degree of nerve compression^a^	172 (87.3)
Degree of intervertebral disc displacement	141 (71.6)
Correlations between level(s) of disc displacement on MRI and clinical symptoms	119 (60.4)
Number and level of displaced discs	55 (27.9)
Degree of intervertebral disc degeneration	31 (15.7)
Vertebral alignment	29 (14.7)

Physical examination	

Spurling test^a^	169 (85.8)
Foraminal compression test	87 (44.2)
Manual muscle testing	72 (36.5)
Adson's test	56 (28.4)
Distraction test	54 (27.4)
Sensory testing	30 (15.2)
Valsalva test	27 (13.2)
Hoffmann's sign	23 (11.7)
Traction test: distraction of arm while taking pulse	14 (7.1)

Korean Medicine syndrome differentiation theories	

Eight-principle pattern identification (八綱辨證)^a^	137 (69.5)
Meridian system diagnosis (經絡辨證)	128 (65.0)
Etiological Factor syndrome differentiation (病因辯證)	95 (48.2)
Qi and Blood diagnosis (氣血辨證)	68 (34.5)
Organ system diagnosis (臟腑辨證)	60 (30.5)
Six meridian diagnoses ( 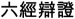 )	44 (22.3)
Sasang constitutional medicine diagnosis (四象體質辨證)	36 (18.3)

Korean Medicine classifications associated with CDH	

Stagnation of Qi and coagulation of blood (氣滯血瘀)^a^	174 (88.3)
Lack and deficiency of liver and kidney + exopathogen (肝腎虧虛+外邪)	105 (53.3)
Deficiency of Qi and blood (氣血虛)	101 (51.3)
Wind-heat with dampness (風熱挾濕)	64 (32.5)
Exuberance of Yang of the liver (肝陽上亢)	53 (26.9)
Wind-dampness exogenous affection (外感風濕)	46 (23.4)

^a^Factor most frequently ranked 1^st^

MRI, magnetic resonance imaging; CDH, cervical disc herniation.

**Table 5:** Frequently prescribed Korean Medicine treatments for cervical disc herniation.

Factors	n (%)
Herbal medicine	

Chungpa-jun (*Eucommia ulmoides* Oliver, *Acanthopanax sessiliflorus* Seem,* Achyranthes japonica* Nakai, *Saposhnikovia divaricata* Schischek, *Cibotium barometz* J. Smith, and *Glycine max* Merrill)^a^	183 (92.89)
Seokyung-tang (舒經湯), Gamiseokyung-tang (加味舒經湯)	166 (84.26)
Galgeun-tang (葛根湯)	51 (25.89)
Gamihwalhyul-tang (加味活血湯)	51 (24.89)
Oyaksoongi-san (烏藥順氣散)	34 (17.26)
Hoesu-san (回首散)	33 (16.75)

Chuna manual medicine	

Supine cervical JS distraction correction technique^a^	163 (82.7)
Supine cervical distraction method using both hands	88 (44.7)
Supine cervical correction technique	70 (35.5)
Prone cervical distraction method	58 (29.4)
Prone both pisiform lower thoracic flexion displacement correction technique	25 (12.7)
Prone anteriorly rotated ilium correction technique	18 (9.1)
Supine cervical distraction method using towel	16 (8.1)
Supine thoracic extension displacement correction technique	16 (8.1)
Side lying lumbar “pitch and roll” distraction method	14 (7.1)
Supine atlanto-correction technique	13 (6.6)
Supine occipital correction technique	13 (6.6)
Prone leg raise ilium correction technique	10 (5.1)

Style of acupuncture	

Ashi points	189 (95.9)
Motion Style Acupuncture Treatment (MSAT)^a^	173 (87.8)
Acupoints relevant to symptoms (acupoints related to specific disorder/syndromes)	105 (53.3)
Dong-Si acupuncture	32 (16.2)
Sa-am acupuncture treatment	25 (12.7)
Five Su (introductory) points	15 (7.6)

Pharmacopuncture	

Shinbaro^a^	160 (81.2)
Bee venom	149 (75.6)
Hwangryunhaedok	126 (64.0)
Joongseongouhyul	58 (29.4)

Acupuncture acupoints	

GB20	150 (76.1)
GB21	127 (64.5)
GV16	56 (28.4)
LI11	51 (25.9)
SI03	49 (24.9)
LI04	40 (20.3)

Pharmacopuncture acupoints	

GB20	147 (74.6)
GB21	131 (66.5)
GV16	61 (31.0)
GB12	18 (9.1)
LI11	16 (8.1)
BL11	11 (5.6)

^a^Factor most frequently ranked 1^st^

**Table 6:** Acupuncture and pharmacopuncture treatment frequently used for cervical disc displacement: data collected and reported according to STRICTA standards.

STRICTA checklist items		Acupuncture			Pharmacopuncture	
Acupuncture rationale	(1a) Style of acupuncture	Refer to Table 4.		(1a) Type of pharmacopuncture	Refer to Table 4.	

	(1b) Reasoning for treatment provided	Anatomical structure likely to cause symptoms (e.g., shortened scalenes and shortened suboccipital muscles)	140 (71.1)			

		Spinal levels of pathology as confirmed through imaging (e.g., site of disc herniation)^a^	118 (59.9)			

		Tender points, trigger points, and other points that elicit a painful response upon palpation	105 (53.3)			

		Ashi points (site of pain)	86 (43.7)			

		Effective acupoints as observed through clinical experience	66 (33.5)			

		Academic knowledge acquired from research articles and clinical practice guidelines	28 (14.2)			

		Acupoints based on Korean Medicine principles (e.g., GB20, GB21, LI11, LI04, and SI03)	28 (14.2)			

		Knowledge acquired through formal education	17 (8.6)			

Details of needling	(2a) Number of needle insertions per subject per session		10.0±3.5	(2a) Number of acupoint injections per subject per session (range)	Anterior	3.6±2.3
					Posterior	6.1±3.6

				(2a) Amount of pharmacopuncture solution injected per session (range, cc)	Anterior	1.1±0.7
					Posterior	2.6±1.9

	(2b) Names of points used	Refer to Table 5.		(2b) Names of points used	Refer to Table 5.	

	(2c) Depth of insertion (cm)		2.1±0.9	(2c) Depth of insertion (range, cm)	Anterior	1.3±0.8
					Posterior	2.7±1.3

	(2d) Responses sought	De qi sensation	5.2±1.4			

		Muscle twitch response	5.1±1.2			

	(2e) Needle stimulation	Motion Style Acupuncture Treatment (MSAT)	111 (56.3)			

		Lifting and thrusting ( 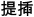 )	75 (38.1)			

		Holding and twisting ( 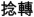 )	74 (37.6)			

		Percentage of patients treated with electroacupuncture (%)	93.5±15.8			

	(2f) Needle retention time (minutes)		13.3±2.5			

	(2g) Needle type	Diameter of needle (mm)	16.1±11.7			

Treatment regimen	(3a) Number of treatment sessions	Refer to Table 2.		(3a) Number of treatment sessions	Refer to Table 2.	

	(3b) Frequency of treatment sessions (sessions/week)		2.1±1.1	(3b) Frequency of treatment sessions (sessions/week)		3.2±7.1

	(3b) Duration of treatment sessions (minutes)		13.3±2.5	(3b) Duration of treatment sessions (range, minutes)	Anterior	2.6±2.9
					Posterior	4.3±4.0

Other components of treatment	(4a) Other interventions administered	Refer to Table 2.		(4a) Other interventions administered	Refer to Table 2.	

Practitioner background	(5) Description of participating acupuncturists	Refer to Table 1.		(5) Description of participating acupuncturists	Refer to Table 1.	

^a^Factor most frequently ranked 1^st^

STRICTA, standards for reporting interventions in clinical trials of acupuncture.

**Table 7:** Perceived safety of Korean Medicine treatments for cervical disc herniation.

Factors	Mean±SD
Cupping	6.4±0.8
Acupuncture	6.3±0.8
Herbal medicine	6.3±0.8
Pharmacopuncture	6.1±0.9
Doin conduction	5.9±1.0
Physiotherapy	5.8±1.0
Qigong, Tai Chi	5.6±1.4
Chuna manual therapy	5.6±1.0
Moxibustion	5.3±1.4
Bee venom pharmacopuncture	4.7±1.3

(Safety: 1=very unsafe 2=unsafe, 3=somewhat unsafe, and 4=not safe, but not unsafe, 5=somewhat safe, 6=safe, and 7=very safe.)

## Data Availability

The data that support the findings of this study are available from the authors upon reasonable request and with permission of the Institutional Review Board of Jaseng Hospital of Korean medicine.

## Supplementary Materials

Supplementary Materials Supplementary PDF file is a translated English language version of the final questionnaire used for collection of data.
