# Supplementary material for: Survey of Integrative Treatment Practices of Korean Medicine Doctors for Cervical Disc Herniation: Preliminary Data for Clinical Practice Guidelines
Source: Evid Based Complement Alternat Med. 2019 Jul 31;2019:2345640. doi: 10.1155/2019/2345640 (PMC6732643; doi:10.1155/2019/2345640)
Supplement: Supplementary Materials — Supplementary PDF file is a translated English language version of the final questionnaire used for collection of data. [file 2345640.f1.pdf]

Clinical practice survey of Korean medicine treatment for cervical disc herniation

[Objectives]

By collecting data and opinions on clinical practice patterns of Korean Medicine treatment of cervical disc herniation, we hope to:

① **Establish evidence for standardization of Korean Medicine treatment**

② **Establish the basis for future clinical practice guidelines on cervical disc herniation**

③ **Investigate the differences between guideline and actual clinical practice**

Clinical practice guidelines (CPGs) are guidelines constructed from evidence presented in the form of randomized controlled trials (RCTs) and systematic reviews (SRs). However, although numerous RCTs and SRs have been conducted, they have yet to cover all disorders and relevant treatment methods, and guidelines synthesized from trials results cannot fully reflect clinical practice. Furthermore, RCTs and SRs are generally labor-intensive and time-consuming requiring much time and resources. Systematic collection of practitioner opinion has been proposed as an alternative method of constructing guidelines. However, there is insufficient information on Korean Medicine physician opinion in treatment of neck pain and cervical disorders, and we hope that the data collected through this survey may form the foundation as basic groundwork for Korean Medicine CPGs on neck pain and cervical disorders.

[Definition of cervical disc herniation]

**Cervical disc herniation**	M50	Cervical disc disorders
	M4722	Other spondylosis with radiculopathy, cervical region
	M4723	Other spondylosis with radiculopathy, cervicothoracic region

Cervical disc herniation includes radiculopathy where cervical disc herniation is not yet confirmed.
The above definition referred to high frequency Health Insurance Review & Assessment Service (HIRA) Standard Classification of Diseases codes, and was reached through discussion with Korean Medicine doctors employed at spine-specialty Korean Medicine hospitals.

[Questionnaire construction process]

This survey was designed and constructed (a) based on a survey that was compiled with reference to 55 published articles on surveys conducted in medical/healthcare providers of spinal disc herniation / spinal stenosis treatment, individually modified by 4 Korean Medicine doctors affiliated with spine-specialty Korean Medicine hospitals, and completed through consultation with 5 extramural experts, and (b) with additional reference to 10 published articles identified through searches on surveys conducted in medical/healthcare providers of cervical disc herniation treatment. Original questionnaires were compiled for a first draft, and the questionnaire was completed through discussions, and supplementations and revisions of the initial draft based on individual modifications by 3 Korean Medicine specialists employed at a Korean Medicine hospital specializing in spinal disorders and comments from 6 extramural medical specialty experts with clinical experience of 10 years or longer. Sample answers provided are based on various references including 『Oriental Rehabilitation Medicine 4th edition (Society of Korean Medicine Rehabilitation)』, 『Chuna Medicine 2nd edition (Korean Society of Chuna Manual Medicine for Spine & Nerves)』, 『Textbook of Acupuncture and Moxibustion (Korean Acupuncture and Moxibustion Medicine Society Textbook

Publication Committee)』, and 『Clinical Guideline on Acupuncture Treatment for Neck Pain (Korean Acupuncture and Moxibustion Medicine Society)』, and additional answer choices were limited to items considered necessary after sufficient discussion.

[Instructions]

While the majority of questions are multiple-choice, some require you to rank items or give short responses. Please feel free to add comments should you feel there is a more appropriate answer.

We appreciate the time and effort taken to participate in this survey. Your answers will contribute to a foundation for standardization of Korean Medicine care for spinal disorders. The contents of this questionnaire will be used in statistical analysis for academic purposes only, and any personal information will remain strictly confidential other than for statistical analyses. In order to ensure anonymity, the action of filling out the survey will be considered to substitute consent to participation instead of obtaining individual study participation consent forms.

Jaseng Spine and Joint Research Institute

Jaseng Medical Foundation

No.	

[PART 1. Demographic information]

1. Age: _____ years old

2. Gender: ① Male ② Female

3. Years of clinical experience (including years of residency, and service as a public health doctor or medical officer): _____ years

4. Under what category is your affiliated institution classified?

① Primary healthcare institution (clinic/hospital with under 30 inpatient beds)

② Secondary healthcare institution (mid-range hospital/conforming to general hospital standards with 30 to 500 inpatient beds)

5. Where is your affiliated institution located?

① Seoul ② Busan ③ Incheon ④ Daegu ⑤ Gwangju ⑥ Daejeon
⑦ Ulsan ⑧ Gyunggi ⑨ Gangwon ⑩ Chungbuk ⑪ Chungnam ⑫ Jeonbuk ⑬ Jeonnam
⑭ Gyeongbuk ⑮ Gyeongnam ⑯ Jeju

6. Do you have any experience practicing at a specialty hospital for spinal disorders as designated by the Korean Ministry of Health and Welfare?

① Currently practicing ② Have practiced in the past ③ Have never practiced

7. Highest academic degree: ① Bachelor's degree ② Master's degree ③ Ph.D.

8. Specialist training: ① Yes (specialist) ② No (general practitioner) ③ Currently in residency (resident)

8-1. Specialty (Check if applicable)

① Korean Medicine Rehabilitation ② Korean Acupuncture and Moxibustion Medicine
③ Oriental Neuropsychiatry ④ Internal Korean Medicine
⑤ Korean Medicine Obstetrics and Gynecology ⑥ Korean Medicine Ophthalmology
⑦ Sasang Constitutional Medicine ⑧ Korean Oriental Pediatrics

9. Extracurricular Korean Medicine or conventional medicine training (received at academic societies, or through certification programs)

① Korea Pharmacopuncture Institute<br>② Korean Medicine Association of Clinical Sanghan-Geumgwe<br>③ Korean Society of Chuna Manual Medicine for Spine & Nerves<br>④ Society of Korean Medicine Pain Control & Delivery System<br>⑤ Korea Immuno-Yakchim Society ⑥ Society of Hyungsang Medicine<br>⑦ Korean Acupuncture and Moxibustion Medicine Society<br>⑧ Society of Korean Medicine Rehabilitation<br>⑨ Korean Academy of Sports Oriental Medicine<br>⑩ Society of Korean Medicine Obstetrics and Gynecology<br>⑪ Other: ______________________________ **[multiple responses allowed]**

※Top 10 Korean Medicine societies in number of members listed in the Society of Korean Medicine as of August 2016

[PART 2. Clinical practice patterns]

1. The following questions concern clinical practice patterns in outpatient care. Please fill in the following blanks to reflect your practice.

**Cervical disc herniation**
A. Average number of outpatients: ______ patients/day
B. Average number of treatment sessions: (   ) times/week
C. Type of intervention:
① Acupuncture ② Pharmacopuncture ③ Bee venom pharmacopuncture
④ Herbal medicine ⑤ Chuna ⑥ Moxibustion ⑦ Cupping
⑧ Physiotherapy (including electrotherapy, light therapy, and hydrotherapy)
⑨ Doin conduction exercise (including instructions for home exercise) ⑩ Qigong, Tai Chi
**[multiple responses allowed]**
D. Average duration of treatment per visit: (   ) minutes
E. Average length of treatment needed for 50% pain decrease: (   ) weeks
F. Average length of treatment needed for 80% pain decrease: (   ) weeks

[PART 3. Tests and Prognostic factors]

1. The following factors are known to influence diagnosis and prognosis of cervical disc herniation patients. Please rate the importance of individual factors on patient prognosis, and in the blank space provided below, rank the factors from most influential. (Mark as A~K)

(Importance: 1=not important at all, 2=unimportant, 3=somewhat unimportant, 4=nether important nor unimportant, 5=somewhat important, 6=important, 7=very important)

Cervical disc herniation	
**Factors**	**Importance**
A. Age	1 2 3 4 5 6 7
B. Past history (e.g. surgery, trauma)	1 2 3 4 5 6 7
C. Time elapsed since onset and cause of onset	1 2 3 4 5 6 7
D. Comorbidities	1 2 3 4 5 6 7
E. Radiological findings	1 2 3 4 5 6 7
F. Clinical symptoms	1 2 3 4 5 6 7
G. Korean Medicine syndrome differentiation	1 2 3 4 5 6 7
H. Physical examination	1 2 3 4 5 6 7
I. Personality and other psychological factors (e.g. depression, anxiety)	1 2 3 4 5 6 7
J. Patient attitude toward and perception of disorder	1 2 3 4 5 6 7
K. Other: ____________________	1 2 3 4 5 6 7
**Rank: 1st ( ) 2nd ( ) 3rd ( )**	

※Reference: 『Clinical Guideline on Acupuncture Treatment for Neck Pain (Korean Acupuncture and Moxibustion Medicine Society)』, articles identified through Oriental Medicine Advanced Searching Integrated System (OASIS) and Korean Traditional Knowledge Portal searches, and expert opinion

2. The following tests are relevant for diagnosis and differential diagnosis in cervical disc herniation patients. Please rank tests in the order of those you most commonly refer to. (Mark as A~Q)

**Cervical disc herniation**
A. Simple X-ray
B. Myelography
C. Discography
D. Computed Tomography (CT)
E. Magnetic Resonance Imaging (MRI)
F. Bone scan
G. Fluoroscopy
H. Digital Infrared Thermal Imaging (DITI)
I. Sonogram
J. Electromyogram
K. C-Reactive protein (CRP)
L. Alkaline Phosphatase (ALP)
M. Creatinine
N. Creatine kinase (CK)
O. Rheumatoid Factor (RF)
P. Erythrocyte Sedimentation Rate (ESR)
Q. Other: ______________________________
**1st (　　)　2nd (　　)　3rd (　　)**

※Reference: 『Clinical Guideline on Acupuncture Treatment for Neck Pain (Korean Acupuncture and Moxibustion Medicine Society)』 and 『Oriental Rehabilitation Medicine, 4th edition (Society of Korean Medicine Rehabilitation)』 pp20-22, 51-52 diagnostic imaging tests, p22-23 clinical pathology tests, p24 Other tests

3. The following questions regard how much you make use of the referred test results. Considering a patient you check for any test results to be a patient "checked", please fill in the following blanks.

3-1. About what proportion of new patients do you check for test results? ___________%

3-2. For about what proportion of returning patients do you check for test results? ___________%

4. Please rank the following points of consideration in the order you consider most important when reading X-rays and MRIs of a cervical disc herniation patient. (Mark as A~J)

Cervical disc herniation
A. Degree of intervertebral disc displacement<br>B. Degree of nerve compression<br>C. Diameter/area of spinal canal<br>D. Number and level of displaced discs (e.g. C2/3 vs. C5/6)<br>E. Degree of intervertebral disc degeneration<br>F. Degree of degeneration of vertebral body and/or joints (spondylosis)<br>G. Correlations between levels of disc displacement on MRI and clinical symptoms<br>H. Vertebral alignment<br>I. Schmorl's nodule<br>J. Other: ________________________________
**1st (　　)　2nd (　　)　3rd (　　)**

5. The following physical examinations are relevant for differential diagnosis in cervical disc herniation patients. Rank the tests from those most commonly applied to reflect your practice. (Mark as A~V)

Cervical disc herniation
A. Soto-Hall test<br>B. Adson's test<br>C. Costoclavicular test<br>D. Wright test<br>E. Traction test – Distraction of arm while taking pulse<br>F. Foraminal compression test<br>G. Distraction test<br>H. Spurling test<br>I. L'Hermitte sign<br>J. Valsalva test<br>K. Vertebral artery patency test<br>L. Examination with stethoscope and palpation of vertebrobasilar artery<br>M. Hoffmann's sign<br>N. Finger escape sign<br>O. Deep tendon reflex<br>P. Spinal percussion test<br>Q. Manual muscle testing<br>R. Sensory testing<br>S. Jackson test<br>T. Brachial plexus test<br>U. Bakody's test<br>V. Other: ________________
**1st (　　)　2nd (　　)　3rd (　　)**

※Reference: 『Clinical Guideline on Acupuncture Treatment for Neck Pain (Korean Acupuncture and Moxibustion Medicine Society)』 and 『Oriental Rehabilitation Medicine, 4th edition (Society of Korean Medicine Rehabilitation)』 pp53-57 and addition of necessary items as decided through discussion with experts

[PART 4. Korean Medicine syndrome differentiation]

1. The following theories are used for Korean Medicine syndrome differentiation. Rank the following theories in the order of most relevant when diagnosing a cervical disc herniation patient. (Mark as A~I)

Cervical disc herniation
A. Eight principle pattern identification (八綱辨證)<br>B. Qi and Blood, Yin and Yang, Body Fluid syndrome differentiation (氣血陰陽津液辨證)<br>C. Organ system syndrome differentiation (臟腑辨證)<br>D. Meridian system syndrome differentiation (經絡辨證)<br>E. Defensive Qi and nutrient Blood syndrome differentiation (衛氣營血辨證)<br>F. Six meridian syndrome differentiation (六經辯證)<br>G. Sasang constitutional medicine syndrome differentiation (四象體質辨證)<br>H. Etiological Factor syndrome differentiation (病因辯證)<br>I. Other: ___________________________
**1st (   )   2nd (   )   3rd (   )**

※Reference: 『Clinical Guideline on Acupuncture Treatment for Neck Pain (Korean Acupuncture and Moxibustion Medicine Society)』, 『Oriental Rehabilitation Medicine, 4th edition (Society of Korean Medicine Rehabilitation)』 p19 Diagnosis and evaluation, and addition of necessary items as decided through discussion with experts

2. Of the following Korean Medicine classifications for neck pain, rank the type(s) you consider to correlate to cervical disc herniation symptoms at a high frequency in order of frequency. (Mark as 1~9)

Cervical disc herniation
① Wind-Dampness Exogenous Affection (外感風濕)<br>② Wind-Heat with Dampness (風熱挾濕)<br>③ Stagnation of Qi and Coagulation of Blood (氣滯血瘀)<br>④ Lack and Deficiency of Liver and Kidney + Exopathogen (肝腎虧虛 + 外邪)<br>⑤ Wind-Cold Exogenous Affection (外感風寒)<br>⑥ Impairment of Body Fluid from Exogenous Heat (邪熱傷津)<br>⑦ Exuberance of Yang of the Liver (肝陽上亢)<br>⑧ Deficiency of Qi and Blood (氣血虛)<br>⑨ Other: ____

※Reference: 『Oriental Rehabilitation Medicine, 4th edition (Society of Korean Medicine Rehabilitation)』 pp84-85 Summary/Etiology and Treatment of Cervical Disorders, 『Clinical Guideline on Acupuncture Treatment for Neck Pain (Korean Acupuncture and Moxibustion Medicine Society)』, 『Dongeuibogam』 Neck part, and addition of necessary items as decided through discussion with experts

[PART 5. Korean Medicine treatment]

1. Grade the following Korean Medicine treatment methods used for cervical disc herniation by how effective each type of treatment is in the short term (8 weeks) / long term (1 year).

(Therapeutic effects: 1=very ineffective, 2=ineffective, 3=somewhat ineffective, 4=neither effective nor ineffective, 5=somewhat effective, 6=effective, 7=very effective)

Cervical disc herniation		
Type of intervention	Treatment effects	
	Short term (8 weeks)	Long term (1 year)
Herbal medicine	1 2 3 4 5 6 7	1 2 3 4 5 6 7
Chuna	1 2 3 4 5 6 7	1 2 3 4 5 6 7
Bee venom	1 2 3 4 5 6 7	1 2 3 4 5 6 7
Pharmacopuncture	1 2 3 4 5 6 7	1 2 3 4 5 6 7
Acupuncture	1 2 3 4 5 6 7	1 2 3 4 5 6 7
Moxibustion	1 2 3 4 5 6 7	1 2 3 4 5 6 7
Cupping	1 2 3 4 5 6 7	1 2 3 4 5 6 7

※Reference: Treatment methods from 『Clinical Guideline on Acupuncture Treatment for Neck Pain (Korean Acupuncture and Moxibustion Medicine Society)』, and OASIS and Korean Traditional Knowledge Portal searches

2. [Acupuncture, Pharmacopuncture] Rank the following acupoint selection rationales in the order of most frequent use when treating cervical disc herniation patients with acupuncture and pharmacopuncture. (Mark as A~I)

Cervical disc herniation
A. Effective acupoints as observed through clinical experience<br>B. Knowledge acquired through formal education<br>C. Academic knowledge derived from research articles, clinical practice guidelines<br>D. Ah-shi points (site of pain)<br>E. Anatomical structure likely to cause symptoms (e.g. shortened scalenes, shortened suboccipital muscles)<br>F. Spinal levels of pathology as confirmed through imaging (e.g. site of disc herniation)<br>G. Tender points, trigger points, and other points that elicit a painful response upon palpation<br>H. Acupoints based on Korean Medicine principles (e.g. GB20, GB21, LI11, LI04, SI03)<br>I. Other: ______________________
**1st (　　) 2nd (　　) 3rd (　　)**

※Reference: Revised STandards for Reporting Interventions in Clinical Trials of Acupuncture (STRICTA): Extending the CONSORT Statement

3. [Acupuncture] The following questions regard acupuncture treatment of cervical disc herniation. Please fill in the blanks for treatments you perform per patient per session. For items on de qi sensation and muscle twitch response, mark the importance of eliciting such response during acupuncture treatment.

(Importance: 1=not important at all, 2=unimportant, 3=somewhat unimportant, 4=not important, not unimportant, 5=somewhat important, 6=important, 7=very important)

	**Cervical disc herniation**
**Names of points used** **[multiple responses allowed]**<br>**(e.g. GB20, GB21, LI11, LI04, SI03, BL62, GB34, LR03, TE05, GV16, BL11, GB12, GV20)***	
**Number of needle insertions**	Average ( ) needles
**Patient position and depth of needle insertion**<br>**(e.g. supine, prone, seated)**	Average ( )cm in ( ) position
**Needle retention time**	Average ( ) minutes
**Diameter of needle**	Average 0. ( )mm
**Needle stimulation** **[multiple responses allowed]**<br>**(e.g. Lifting and thrusting (提揷), Holding and twisting (捻轉), Motion Style Acupuncture Treatment)**	
**Percentage of patients treated with electroacupuncture**	About ( )%
	**Importance**
**How important do you think de-qi sensation is in acupuncture treatment?**	1 2 3 4 5 6 7
**How important do you think muscle twitch responses are in acupuncture treatment?**	1 2 3 4 5 6 7

*Reference: 『Clinical Guideline on Acupuncture Treatment for Neck Pain (Korean Acupuncture and Moxibustion Medicine Society)』

4. [Acupuncture] Rank the following styles of acupuncture in the order you consider to be most effective for treatment of cervical disc herniation. (Mark as A~M)

Cervical disc herniation
A. Ashi points<br>B. Acupoints relevant to symptoms (acupoints related to specific disorder/syndromes)<br>C. Mu-ja Acupuncture (acupuncture on contralateral side)<br>D. Five Element Acupuncture<br>E. Constitution Acupuncture<br>F. Burning Acupuncture (火針)<br>G. Sa-am Acupuncture Treatment<br>H. Dong-Si Acupuncture Treatment<br>I. Motion Style Acupuncture Treatment (MSAT)<br>J. Five Su (Inductory) Points<br>K. Specalized Points (Lower Confluent Points, Source/Connecting/Cleft/Front Mo Points, Eight Influential Points, Eight Confluent Points)<br>L. Taegeuk Acupuncture<br>M. Other: ________________________
**1st (   )   2nd (   )   3rd (   )**

※Reference: Selected from『Clinical Guideline on Acupuncture Treatment for Neck Pain (Korean Acupuncture and Moxibustion Medicine Society)』 and『Textbook of Acupuncture and Moxibustion (Korean Acupuncture and Moxibustion Medicine Society Textbook Publication Committee)』

5. [Pharmacopuncture] The following questions concern pharmacopuncture treatment for cervical disc herniation. Please fill in the blanks for treatments you perform per patient per session.

	**Cervical disc herniation**
**Most commonly used types of pharmacopuncture**<br>(e.g. Joongseongouhyul, Bee Venom Pharmacopuncture, Carthami Flos. Pharmacopuncture, Soyeom Pharmacopuncture, Hwangryunhaedok-tang Pharmacopuncture, Shinbaro Pharmacopuncture)*	**1st ( )**<br>**2nd ( )**<br>**3rd ( )**
**Names of points used** **[multiple responses allowed]**<br>(e.g. GB20, GB21, LI11, LI04, SI03, BL62, GB34, GV16, BL11, GB12, GV20)*	
**Length of needle**	( ) cm ~ ( ) cm
**Number of acupoint injections per session**	( ) points ~ ( ) points
**Amount of pharmacopuncture solution injected per session**	( ) cc ~ ( ) cc
**Duration of treatment sessions**	( ) minutes ~ ( ) minutes
**Frequency of treatment sessions**	( ) sessions/week

*Reference:『Clinical Guideline on Acupuncture Treatment for Neck Pain (Korean Acupuncture and Moxibustion Medicine Society)』 and PUBMED research articles

6. **[Herbal medicine] The following questions concern herbal medicine treatment of cervical disc herniation. Rank the following herbal prescriptions in order of most effective. (Mark as A~O)**

**Cervical disc herniation**
A. Shintongchuguh-tang (身痛逐瘀湯)<br>B. Gamihwalhyul-tang (加味活血湯)<br>C. Oyaksoongi-san (烏藥順氣散)<br>D. Hoesu-san (回首散)<br>E. Seokyung-tang (舒經湯), Gamiseokyung-tang (加味舒經湯)<br>F. Ssanghwa-tang (雙和湯)<br>G. Daeboeum-hwan (大補陰丸)<br>H. Yookmijihwang-tang (六味地黃湯)<br>I. Bojoongikgi-tang (補中益氣湯)<br>J. Chungpa-jun (Eucommia ulmoides Oliver, Acanthopanax sessiliflorus Seem, Achyranthes japonica Nakai, Saposhnikovia divaricata Schischk, Cibotium barometz J. Smith, Glycine max Merrill)<br>K. Galgeun-tang (葛根湯)<br>L. Ganghwalseungseup-tang (羌活勝濕湯)<br>M. Mangeum-tang (萬金湯)<br>N. Youngsunjetong-eum (靈仙除痛飮)<br>O. Other: ________________________
**1st (　　)　2nd (　　)　3rd (　　)**

※Reference: 『Oriental Rehabilitation Medicine 4th edition (Society of Korean Medicine Rehabilitation)』 p85 frequently used prescriptions for cervical disorders, relevant research articles, and addition of necessary items as decided through discussion with experts

7. [Chuna] The following Chuna manipulation techniques are relevant to cervical disc herniation. Rank the techniques from those most commonly used in your practice. (Mark as A~l)

Cervical disc herniation

Cervical spine

A. Supine cervical distraction method using towel
B. Supine cervical distraction method using both hands
C. Prone cervical distraction method
D. Supine cervical correction technique
E. Supine cervical JS distraction correction technique
F. Supine atlanto-correction technique
G. Supine occipital correction technique

Thoracic spine and rib cage

H. Sitting, upper thoracic extension displacement muscle release/reinforcement technique
I. Sitting, upper thoracic flexion displacement muscle release/reinforcement technique
J. Sitting, upper thoracic neutral dysfunction muscle release/reinforcement technique
K. Supine thoracic extension displacement correction technique
L. Prone both pisiform lower thoracic flexion displacement correction technique
M. Sitting, lower thoracic extension displacement muscle release/reinforcement technique
N. Sitting, lower thoracic flexion displacement muscle release/reinforcement technique
O. Sitting, lower thoracic neutral dysfunction muscle release/reinforcement technique
P. Sitting, 1st rib superior displacement muscle release/reinforcement technique
Q. Rib cage Fascia Chuna technique: Supine 2nd rib lateral flexion displacement joint mobilization technique

Lumbar spine

R. Prone lumbosacral joint distraction method
S. Sidelying lumbar 'pitch and roll' distraction method
T. Sidelying lumbar extension displacement correction technique
U. Sidelying lumbar flexion displacement correction technique
V. Sidelying lumbar neutral dysfunction correction technique
W. Sitting, lumbar bilateral flexion displacement muscle release/reinforcement technique
X. Spine flexion distraction method: Flexion shift technique
Y. Spine flexion distraction method: Sidelying technique
Z. Spine flexion distraction method: Circumduction method
a. Spine flexion distraction method: Magnum circulation technique
b. Spine flexion distraction method: Extension technique

Ilium

c. Prone leg raise ilium correction technique
d. Prone anteriorly rotated ilium correction technique
e. Prone pisiform, metacarpophalangeal joint of 2nd finger inflare-outflare correction technique
f. Prone posteriorly rotated ilium/sidebent sacrum correction technique
g. Sidelying ilium correction technique

Sacrum

h. Prone sacrum flexion displacement correction technique
i. Prone sacrum extension displacement correction technique
j. Prone sacrum sidebent rotation displacement correction technique
k. Sidelying sacrum correction technique

Other

l. (   )

1st (   )   2nd (   )   3rd (   )

※Reference: 『Chuna Medicine, 2nd edition (Korean Society of Chuna Manual Medicine for Spine & Nerves)』

[PART 6. Safety]

1. Rank how safe you consider the following interventions to be for cervical disc herniation.

(Safety: 1=very unsafe 2=unsafe, 3=somewhat unsafe, 4=not safe, but not unsafe, 5=somewhat safe, 6=safe, 7=very safe)

Type of intervention	Safety
**Acupuncture**	1 2 3 4 5 6 7
**Pharmacopuncture**	1 2 3 4 5 6 7
**Bee venom**	1 2 3 4 5 6 7
**Chuna**	1 2 3 4 5 6 7
**Herbal medicine**	1 2 3 4 5 6 7
**Cupping**	1 2 3 4 5 6 7
**Moxibustion**	1 2 3 4 5 6 7
**Physiopherapy**	1 2 3 4 5 6 7
**Doin conduction**	1 2 3 4 5 6 7
**Qigong, Tai Chi**	1 2 3 4 5 6 7

2. Rank the following interventions in order of most likely cause of adverse effect(s) in cervical disc herniation, and select potential adverse event(s) for each type of treatment. **[multiple responses allowed]**

(e.g. 1st Type of intervention (C) – Adverse effect (1, 3, 4, 5, 15))

Type of intervention	Adverse events
A. Acupuncture<br>B. Pharmacopuncture<br>C. Bee venom<br>D. Chuna<br>E. Herbal medicine<br>F. Cupping<br>G. Moxibustion<br>H. Physiopherapy<br>I. Doin conduction<br>J. Qigong, Tai Chi	① Headache<br>② Abdominal pain<br>③ Allergic reactions including pruritus and rashes<br>④ Anaphylaxis<br>⑤ Aggravation of pre-existing pain<br>⑥ Gastrointestinal disorders<br>⑦ Urination/defecation dysfunction<br>⑧ Infection<br>⑨ Bleeding and vascular injury<br>⑩ Nerve injury<br>⑪ Amyotrophy<br>⑫ Muscle/tendon/ligament tear<br>⑬ Organ injury (e.g. needle penetrating kidney, intestines)<br>⑭ Altered consciousness accompanied by vital sign change<br>⑮ Pneumothorax<br>⑯ Other adverse events: ________________________
**1st Type of intervention ( ) – Adverse event ( )**<br>**2nd Type of intervention ( ) – Adverse event ( )**<br>**3rd Type of intervention ( ) – Adverse event ( )**	
